# Mast cells are essential in the development of exposure-associated exercise-induced bronchoconstriction in a mouse model

**DOI:** 10.3389/fimmu.2025.1650057

**Published:** 2025-10-15

**Authors:** Nora F. Marain, Fien Byttebier, Marieke Colemont, Ellen Dilissen, Jonathan Cremer, Dominique MA Bullens, Lieven J. Dupont, Jeroen A. Vanoirbeek

**Affiliations:** ^1^ Department of Chronic Diseases and Metabolism, Laboratory of Respiratory Diseases and Thoracic Surgery, Katholieke Universiteit (KU) Leuven, Leuven, Belgium; ^2^ Department of Microbiology, Immunology and Transplantation, Allergy and Clinical Immunology Research Group, Katholieke Universiteit (KU) Leuven, Leuven, Belgium; ^3^ Clinical Division of Paediatrics, UZ Leuven, Leuven, Belgium; ^4^ Clinical Division of Respiratory Medicine, UZ Leuven, Leuven, Belgium; ^5^ Department of Public Health and Primary Care, Environment and Health, Katholieke Universiteit (KU) Leuven, Leuven, Belgium

**Keywords:** mast cells, exercise-induced bronchoconstriction, cold air, diesel exhaust particles, doxantrazole

## Abstract

**Background:**

Cold air and air pollution are known triggers to induce symptoms in exercise-induced bronchoconstriction (EIB). Mast cells are hypothesized to be a key player in the pathogenesis of EIB. This study aims to investigate the role of mast cells using mast cell deficient (Cpa3^cre/+^) mice and with mast cell stabilizers (Doxantrazole) in an exposure-associated mouse model of EIB.

**Methods:**

Male Cpa3^cre/+^ mice and wild type littermates or BALB/c mice were exposed to a submaximal running protocol in cold environment (4°C) or resting period (room temperature) 5 days for 3 weeks after oropharyngeal challenge with saline or 0.1 mg/ml diesel exhaust particles (DEP). BALB/c mice were intraperitoneally injected with 16.5 mg/kg Doxantrazole or placebo (0.5% NaHCO_3_) during the last week. Twenty-four hours after the last running or resting session, lung function, lung inflammation and immune mediated response was determined.

**Results:**

Inhibition of mast cells by Doxantrazole or mice lacking functional mast cells (Cpa3^cre/+^), resulted in blunting of bronchial hyperreactivity, both in acute breathing pattern and in hyperreactivity to increasing doses of methacholine. Neutrophilic inflammation was still present after Doxantrazole treatment, but not in Cpa3^cre/+^ mice. These results were similar in neutrophil extracellular traps and neutrophil-linked cytokines and chemokines. Macrophage activaty was also reduced in the absence of functional mast cells.

**Conclusion:**

Mast cells are crucial in the development of bronchial hyperreactivity and macrophage activation. Additionally, they have a complex interplay with neutrophilic inflammation. These findings highlight the potential of mast cell modulation as therapeutic strategy in exposure-associated EIB.

## Introduction

Exercise-induced bronchoconstriction (EIB) is defined as the reversible and transient narrowing of the airways during or shortly after physical exertion (1). Typically experienced symptoms include cough, chest tightness, shortness of breath and wheezing. Although the prevalence is high in both athletes and patients with asthma, with an average of 30 – 70% and 40 – 90%, respectively, the pathophysiological mechanisms are still not fully understood (2).

Couto et al. suggests that mast cells are a key player in EIB, next to neutrophils and TRP-channels (3). Mast cells are bone-marrow derived cells that mature under the influence of stem cell factor, the ligand of the c-kit receptor. Mast cell precursors migrate from the bone marrow to epithelial and mucosal tissues, such as the gastrointestinal tract, skin and the respiratory epithelium, where they complete maturation (4). Different subtypes of mast cells have been identified in both humans and mice, distinguished by their granule content, tissue localisation and functional characteristics (5).

In the lung, mast cells can promote airway smooth muscle contraction through the release of contractile agonists such as histamine and cysteinyl leukotriene (6). While IgE/FcϵRI-dependent mast cell activation is most studied and understood (7), IgE/FcϵRI-independent routes also contribute to bronchoconstriction. The mast cell receptor MAS-related G protein-coupled receptor X2 (MRGPRX2) was over 15 years ago identified in humans to play an important role in IgE-independent mast cell activation (8, 9). Although several studies report increased numbers of MRGPRX2-positive mast cells in lung tissue from patients with asthma (10, 11), its exact role in the pathogenesis of asthma remains incompletely understood and difficult to explore (8). Thus, MRGPRX2 may contribute to mast cell–mediated airway responses under certain conditions, but its precise role in asthma pathogenesis requires further clarification.

Different mouse models lacking functional mast cells have been developed and used to study allergic and non-allergic asthma (12–15). Kit^W-sh/W-sh^ mice have the W-sash inversion mutation in the regulatory elements upstream of the c-kit transcription site (16), whereas the Cpa3^cre/+^ mice exhibit mast cell deficiency as a result of Cre-mediated toxicity driven by the carboxypeptidase A3 promotor (17). Both models lack mast cells in all tissues, while maintaining normal levels of other immunological cells, such as neutrophils and erythrocytes (13, 18). Cpa3^cre/+^ mice have an additional reduction in the number of basophils compared to wild type mice (18).

In this study, we aim to evaluate mast cell involvement in the development of bronchial hyperresponsiveness (BHR) and airway inflammation in an EIB-mouse model exposed to cold air and/or diesel exhaust particles (DEP). To achieve this, mice lacking functional mast cells (Cpa3^cre/+^ mice) and wild type (WT) littermates were compared. Additionally, therapeutic inhibition of BHR and inflammation was evaluated with a mast cell stabilizer [doxantrazole (19)] in WT BALB/c mice.

## Methods

### Reagents

Diesel exhaust particles (DEP, NIST2975), which was characterized by the National Institute of Standards and Technology (NIST), Tween^®^20 and doxantrazole (3-(1H-tetrazol-5-YL)-9H-thioxanthen-9-one 10,10-dioxide monohydrate) were obtained from Merck (Belgium). Doxantrazole was dissolved in 0.5% NAHCO_3_ and sonicated for 25 minutes. Pentobarbital (Dolethal^®^) and Isoflurane (Iso-Vet 1000 mg/g) were obtained via the animal facility from KU Leuven.

### Animals

Eight- to ten-week old male BALB/c mice (n = 80) were obtained from Charles River Laboratories (Belgium). Cpa3^Cre/+^ gene-targeted mice were kindly provided by Prof. Hans-Reimer Rodewald (17) and were bred on a BALB/c background. Cpa3^cre/+^ mice (n = 64) and wild type littermates (n = 53) were used in the experimental set-up. To ensure an adequate sample size, littermates were combined with wild type BALB/c mice (Charles River Laboratories, n = 64) after an evaluation of potential differences between experiments. Schematic overview of the experiments can be found in **Supplementary Figure S1**.

Mice were housed in individually ventilated cages per experimental group with *ad* libitum access to food and water. Environmental conditions were standardized (12h dark/light cycles, 22 – 24°C and relative humidity of 50-60%). All experiments were approved by the local Ethical Committee for animal experiments of KU Leuven, Belgium (P118/2021) and comply with the ARRIVE guidelines.

### Experimental running and instillation protocol

A previously optimized sub-maximal running protocol of 3 weeks in combination with DEP and cold exposure was used to induce EIB as described earlier (20, 21). Immediately before each running or resting session, animals were oropharyngeally instilled with 50 µl 0.1 mg/ml DEP, dissolved in vehicle (saline + 0.2% Tween20) or with 50 µl vehicle.

Mice were divided into 8 groups based on 3 variables (1): Exercise (E) or No Exercise (NE) (2); Room temperature (RT) or cold air (4°C) and (3) DEP or Saline (Sal). During the first four days of the last week of the protocol, mice in the mast cell stabilizer experiment were intraperitoneally injected with doxantrazole (16.5 mg/kg) or placebo (50 µl NaHCO_3_) one hour before oropharyngeal instillation with saline or DEP. In this experimental set-up, only RT resting mice (NE/RT) and 4°C exercising (E/4°C) mice were evaluated, with saline or DEP.

### Lung function assessment

Every first day of the week, respiratory pattern was measured non-invasively using Double Chamber Plethysmography (DCP, Emka Technologies, Paris, France) in conscious mice as described earlier (22).

Lung function measurements were performed 24 hours after the final running session, following the methodology previously described (17, 18) using the flexiVent FX system (SCIREQ, Montreal, Canada, version 7.6) equipped with a Negative Pressure Forced Expiration (NPFE) and FX1 module. Baseline lung function was evaluated using deep inflation (inspiratory capacity), Quick-Prime 3 (QP3) pertubations (airway resistance (Rn), tissue damping (G), tissue elastance (H)) and NPFE pertubations (forced volumes). Subsequently, airway resistance and forced expiratory volume in 0.1 seconds (FEV_0.1_) were measured in response to increasing doses of nebulized methacholine (baseline, 0, 2.5, 5, 10, 20 and 40 mg/ml).

### Blood analysis

Immediately after lung function assessment, an overdose of pentobarbital was administered and blood was collected from the retro-orbital plexus. Blood was centrifuged (1400g, 4°C, 10 min), serum was collected and stored at -80°C until further analyses. Serum surfactant protein D (SpD) was determined using the Mouse SP-D DuoSet ELISA D DuoSet ELISA (R&D Systems, Minneapolis, US, detection limit 62.5 µg) according to manufacturer’s instructions.

### Bronchoalveolar lavage fluid analyses

Bronchoalveolar lavage fluid (BALF) was collected by three washes of the lungs with 700 µl saline (0.9% NaCl). Pooled fluid was centrifuged (1000 g, 10 min, RT) and resuspended cells were counted using an automated cell counter (NanoEnTek, Seoul, Korea). Cytospins were made (300 g, 6 min, Cytospin 3, Shandon, TechGen, Zellik, Belgium) and stained with the Diff-Quick method (ThermoFisher Scientific, Massachusetts, US). 250 cells per slide were counted to determine the proportion of neutrophils, macrophages, eosinophils and lymphocytes.

DEP-uptake in macrophages was evaluated as percentage of loaded macrophages, determined by manually counting 250 cells. The DEP-covered area within the macrophage was calculated using the ImageJ software (NIH, Maryland, US) following the method of Bai et al. (23). Twenty-five macrophages per mouse were manually delineated, DEP-covered area was calculated by the software.

Cytokine and chemokine concentrations of granulocyte-macrophage colony-stimulating factor (GM-CSF), tumour necrosis factor-α (TNF-α), interleukin (IL)-1β, IL-2, IL-4, IL-6, IL-10, IL-33, keratinocyte-derived chemokine (KC) and monocyte chemoattractant protein-1 (MCP-1) were measured using a U-plex Assay (Meso Scale Diagnostics, Maryland, USA) according to manufacturer’s instructions. Neutrophil elastase concentration (Mouse Neutrophil Elastase/ELA2 DuoSet ELISA, R&D Systems, Minneapolis, US, detection limit 12.5 pg/ml) and dsDNA (Quant-iT PicoGreen dsDNA assay kit, ThermoFisher Scientific, Massachusetts, USA) were determined according to manufacturer’s instructions.

### Flow cytometric analyses

Left lung was collected during harvest at the end of the protocol and processed to a single cell suspension. Cells were stained for viability and labelled with different anti-mouse fluorochrome-conjugated monoclonal antibodies to identify dendritic cell as previously described (22). Scatter plots of dendritic cell subpopulations are available in **Supplementary Figure S2**. Overview of used markers for flow cytometric analyses can be found in **Supplementary Table S1**.

### Data analysis

Data are shown as individual mice and group mean or as mean with standard deviation (SD). Normality of the data was assessed using Shapiro-Wilk test. Non-parametric Wilcoxon matched-pairs rank test and parametric unpaired t-test were used to compare two groups. Intergroup differences were evaluated using a one-way parametric ANOVA combined with a Bonferroni multiple comparison *post hoc* test or a non-parametric Kruskal-Wallis test with a Dunn’s multiple comparison *post hoc* test. To evaluate combination effects, two-way ANOVA with a Bonferroni multiple comparison *post hoc* test was used. Data was evaluated using GraphPad Prism (9.3.0, GraphPad Software Inc., San Diego, USA). A level of p < 0.05 was considered significant. To enhance clarity, we restricted group comparisons to RT resting groups (NE/RT) and 4°C exercising groups (E/4°C). Additional groups were included to maintain consistency in the experimental model; however, no comparisons were made between WT and Cpa3^cre/+^ mice in this context. The same strategy and groups were used to compare the data of the doxantrazole experiment.

## Results

### Mast cells play a major role in breathing pattern and lung function

**Figure 1:**
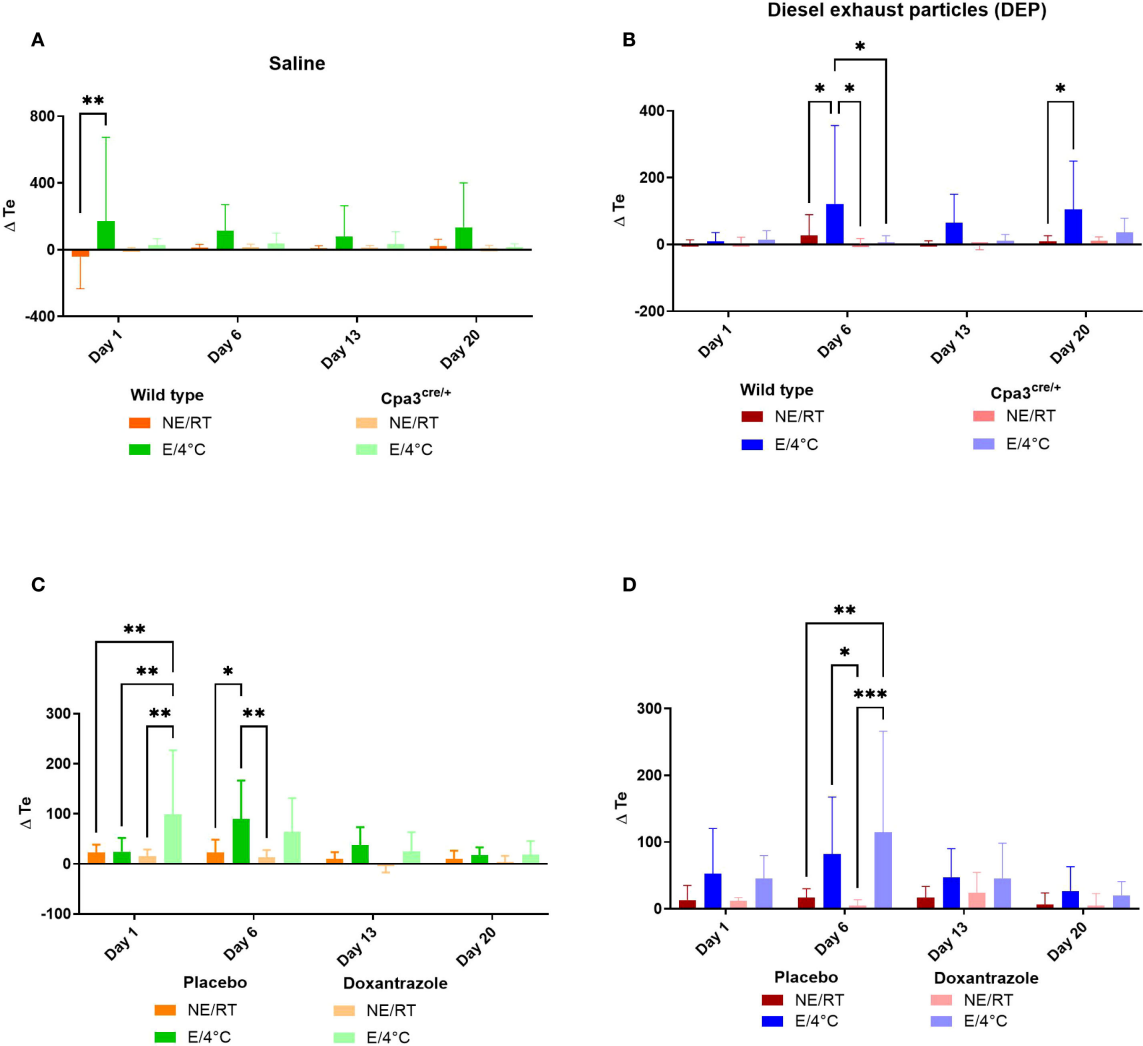
Role of mast cells in responses to cold air, exercise and DEP exposure on expiratory time. Expiratory time (Te) was measured on day 1, 6, 13 and 20 before and immediately after exercise or resting session with double chamber plethysmography, as outlined in **Supplementary Figure S1**. Changes were calculated as differences between pre- and post-exposure. This was assessed in saline-exposed wild type (WT) compared to mast cell deficient (Cpa3^cre/+^) mice **(A)**, in diesel-exposed WT compared Cpa3^cre/+^ mice **(B)**, in saline-exposed placebo-treated mice compared to doxantrazole-treated mice **(C)** and in diesel-exposed placebo-treated mice compared to doxantrazole-treated mice **(D)**. Data are evaluated using two-way ANOVA with Bonferroni multiple comparison *post hoc* test and represented as mean ± SD. Levels of significance were *p < 0.05, **p < 0.01, ***p < 0.001. n = 7–8 per group. NE, no exercise; E, exercise; RT, room temperature.

Previously, we showed significantly changes in the breathing pattern of animals exercising in cold environment (E/4°C) (20). One of the parameters which was significantly increased in the E/4°C groups, was the expiratory time (Te). Mice lacking functional mast cells (Cpa3^cre/+^) did not show an increased Te after exercise at 4°C, both when challenged with saline (**Figure 1A**) and with diesel exhaust particles (DEP, **Figure 1B**). WT DEP cold running mice showed a significantly more increased Te compared to Cpa3^cre/+^ mice. Wild type (WT) mice in the doxantrazole experiment all showed a significantly increased Te compared to non-exercising room temperature (RT/NE) mice (**Figures 1C, D**). The effect of doxantrazole could not be assessed in this setting due to the study design, in which mast cells were only inhibited during the final days. However, this indicates that the mice in the doxantrazole experiment also developed an altered breathing pattern resembling EIB in the first weeks of exercise at 4°C.

**Figure 2:**
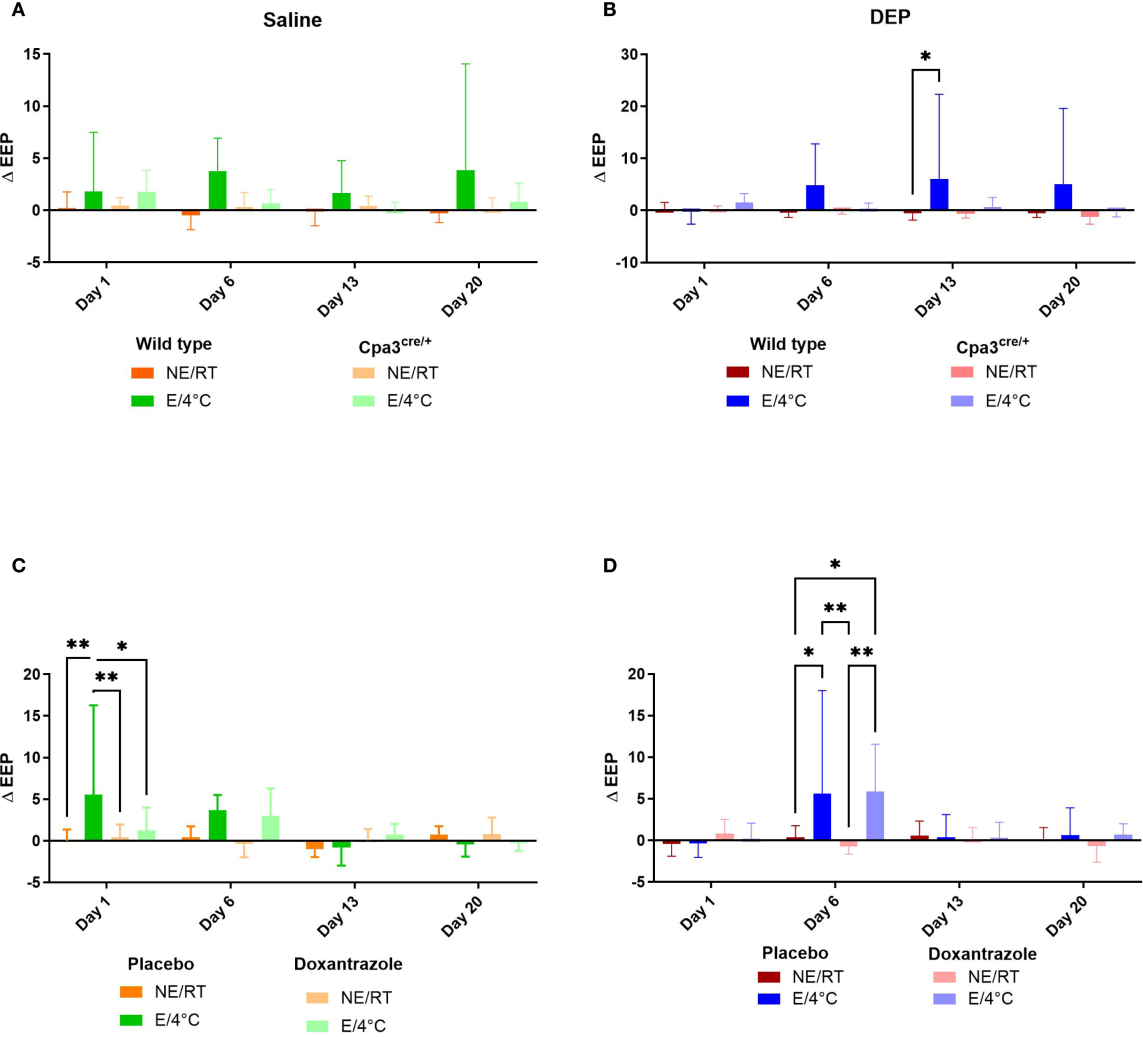
Role of mast cells in responses to cold air, exercise and DEP exposure on end-expiratory pause. End-expiratory pause (EEP) was measured on day 1, 6, 13 and 20 before and immediately after exercise or resting session with double chamber plethysmography based on the experimental protocol (**Supplementary Figure S1**). Changes were calculated as differences between pre- and post-exposure. This was measured in saline-exposed wild type (WT) compared to mast cell deficient (Cpa3cre/+) mice **(A)**, in diesel-exposed WT compared Cpa3cre/+ mice **(B)**, in saline-exposed placebo-treated mice compared to doxantrazole-treated mice **(C)** and in diesel-exposed placebo-treated mice compared to doxantrazole-treated mice **(D)**. Data are evaluated using two-way ANOVA with Bonferroni multiple comparison *post hoc* test and represented as mean ± SD. Levels of significance were *p < 0.05, **p < 0.01, ***p < 0.001. n = 7–8 per group. NE, no exercise; E, exercise; RT, room temperature.

Similar changes were seen in end-expiratory pause (EEP). The increase in EEP in cold air exercising mice exposed to DEP (DEP/E/4°C) as seen in WT animals was no longer present in Cpa3^cre/+^ mice (**Figure 2B**). Similar trends were seen after saline challenge, yet not significant (**Figure 2A**). In the doxantrazole experiment, the mice of the Sal/E(/4°C) also exhibited an increased EEP on day 1 (**Figure 2C**) and mice of the DEP/E(/4°C) group showed an increased EEP on day 6 (**Figure 2D**).

Twenty-four hours after the last running or resting session, lung function was measured invasively. No differences between WT and Cpa3^cre/+^ saline resting RT mice were seen in baseline volumes (FVC, FEV_0.1_, FEV_0.2_), baseline airway resistance (Rn), tissue elastance (H) or tissue damping (G) (data not shown). There were also no baseline differences between the groups in the Cpa3^cre/+^ experiment (data not shown).

**Figure 3:**
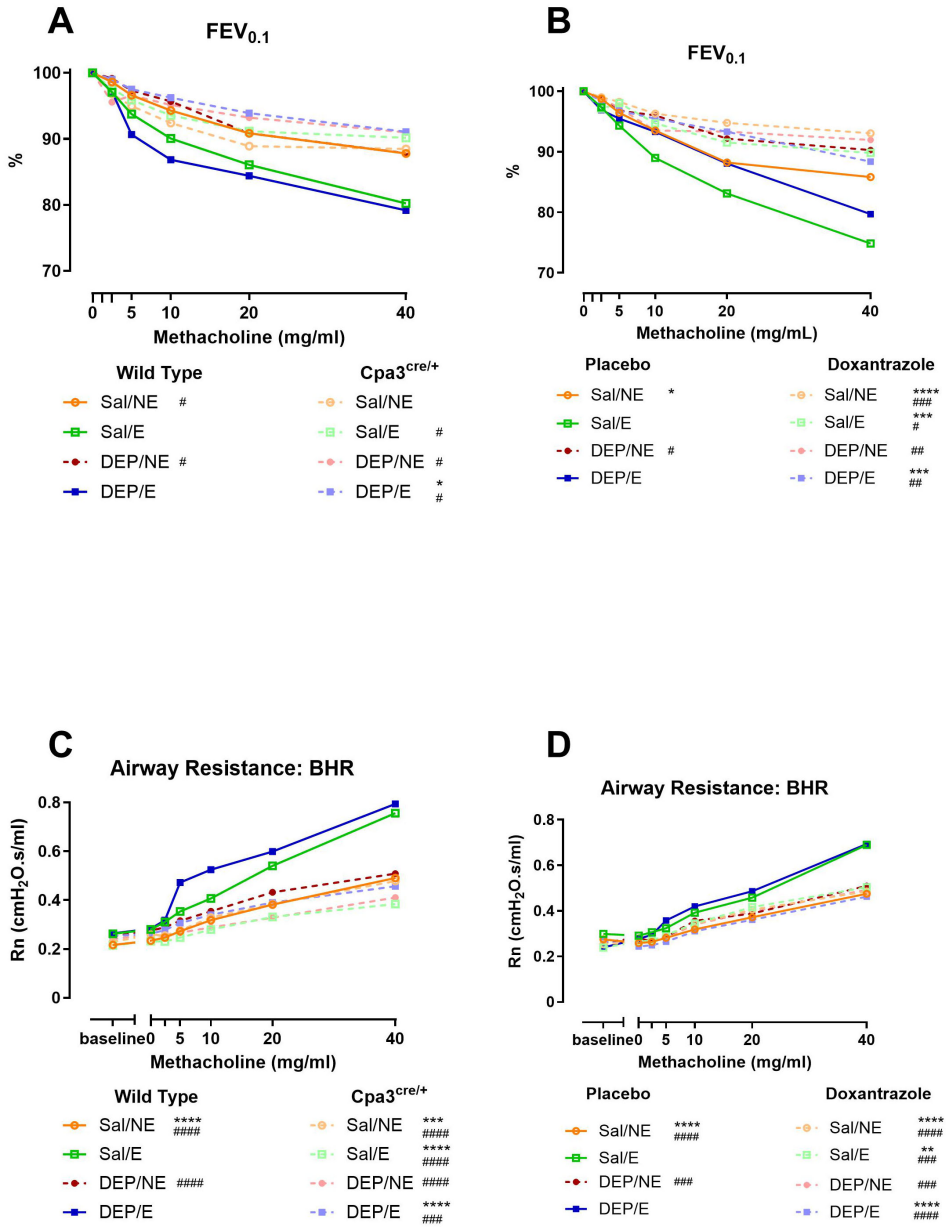
Role of mast cells in bronchial hyperreactivity to cold air, exercise and DEP exposure. Twenty-four hours after the last running or resting session (**Supplementary Figure S1**), animals were sacrificed and reactivity to methacholine was measured using the flexiVent. Dose-response curve in FEV_0.1_ (%) to increasing doses of methacholine (0–40 mg/ml) in WT compared to Cpa3^cre/+^ mice **(A)** and in placebo- compared to Doxantrazole-treated mice **(B)**. Dose-response curve in airway resistance (Rn) to increasing doses of methacholine (0–40 mg/ml) in WT compared to Cpa3^cre/+^ mice **(C)** and in placebo- compared to Doxantrazole-treated mice **(D)**. Data was acquired through negative pressure forced expiration (NPFE) manoeuvre and QP3 forced oscillation technique, respectively, with the flexiVent. Data are represented as group average and evaluated using two-way ANOVA with Bonferroni multiple comparison *post hoc* test, n = 7–16 per group. Levels of significance for groups were * compared to Sal/E (4°C, WT [A&C] or placebo [B&D] and # compared to DEP/E (4°C, WT [A&C] or placebo [C&D]). *, #p < 0.05, **, ##p < 0.01, ***, ###p < 0.001, ****, ####p < 0.0001. n = 7–8 per group. Sal, saline; DEP, diesel exhaust particles; NE, no exercise; E, exercise; RT, room temperature; FEV_0.1_, forced expiratory volume in 0.1 second; BHR, bronchial hyperresponsiveness.

To investigate the role of mast cells in exercise-induced bronchoconstriction in response to cold air, we assessed the reduction in forced expiratory volume in 0.1 second (FEV_0.1_) to increasing doses of methacholine. In WT mice, exercise in cold air induced a dose-dependent decrease in FEV_0.1_, whereas this response was absent in Cpa3^cre/+^ mice (**Figure 3A**). The reduction in FEV_0.1_ was significantly more pronounced in WT cold exercising mice compared to Cpa3^cre/+^ cold exercising mice. No differences were seen between the Cpa3^cre/+^ mice groups (**Supplementary Figure S3A**). One week of doxantrazole treatment was sufficient to inhibit the bronchoconstrictive effect, resulting in a significant lower reduction in FEV_0.1_ compared to placebo (**Figure 3B**). The dose response curves for airway resistance (Rn) showed similar results as the ones for FEV_0.1_ in both experiments, with no significantly increased airway resistance upon methacholine challenge in either Cpa3cre/+ exercising mice (**Figure 3C**, **Supplementary Figure S3B**) or doxantrazole-treated exercising mice (**Figure 3D**).

### Neutrophilic airway inflammation is not completely inhibited by mast cell inhibition

**Figure 4:**
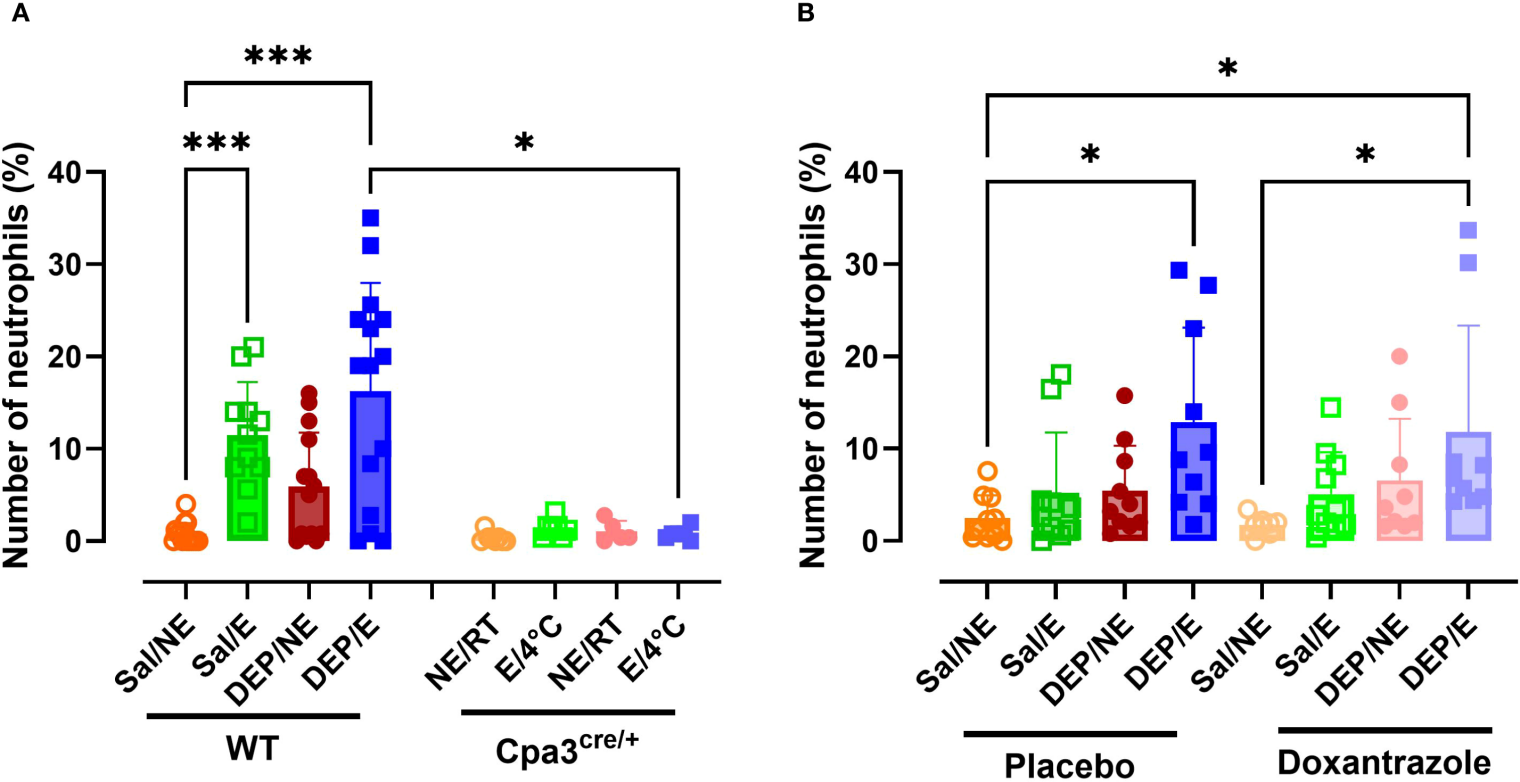
Response of mast cell inhibition on neutrophilic inflammation in the bronchoalveolar lavage fluid. After the experimental running protocol (**Supplementary Figure S1**), animals were sacrificed and bronchoalveolar lavage fluid (BAL) was collected to evaluate (neutrophilic) inflammation. Percentual numbers of neutrophils among total inflammatory cells present in the BAL of Cpa3^cre/+^ mice compared to WT mice **(A)** and placebo- compared to Doxantrazole treated mice. Data are represented as mean ± SD with individual values and were evaluated using Kruskal–Wallis test with Dunn’s *post hoc* testing. Levels of significance were Levels of significance were *p < 0.05, ***p < 0.001. n = 7–16 per group. Sal, saline; DEP, diesel exhaust particles; NE, no exercise; E, exercise; RT, room temperature; WT, wild type.

WT cold exercising mice showed significantly more neutrophils in the bronchoalveolar lavage fluid (BALF) compared to resting (RT) mice, with a more pronounced effect in DEP-challenged animals. This effect was entirely absent in Cpa3^cre/+^ mice. A significantly lower percentage of neutrophils was present in Cpa3^cre/+^ DEP/E/4°C mice compared to WT DEP/E/4°C mice (**Figure 4A**). Additionally, no differences were seen between the different Cpa3^cre/+^ groups (**Supplementary Figure S4**).

Both placebo and doxantrazole treated DEP/E/4°C mice showed significantly higher percentages of neutrophils in the BALF compared to Sal/NE/RT mice (**Figure 4B**). No differences were observed between doxantrazole and placebo treated animals.

**Figure 5:**
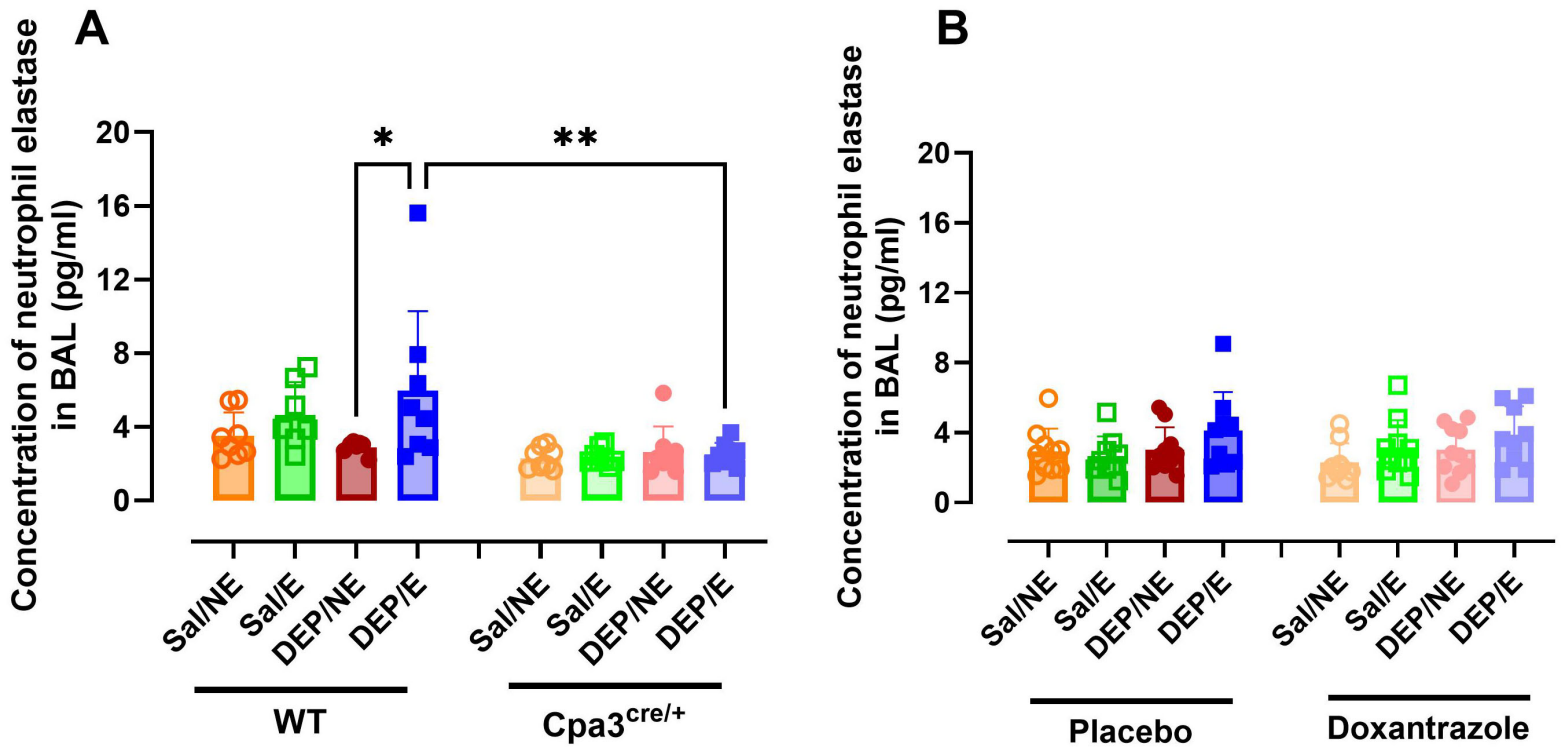
Response of mast cell inhibition on neutrophil extracellular traps in the bronchoalveolar lavage fluid. After the experimental running protocol (**Supplementary Figure S1**), animals were sacrificed and bronchoalveolar lavage fluid (BAL) was collected to evaluate presence of neutrophil extracellular traps (NETs, measured as neutrophil elastase). Concentration of neutrophil elastase present in the BAL of Cpa3^cre/+^ mice compared to WT mice **(A)** and placebo- compared to Doxantrazole treated mice **(B)**. Data are represented as mean ± SD with individual values and were evaluated using Kruskal–Wallis test with Dunn’s *post hoc* testing. Levels of significance were *p < 0.05, **p < 0.01. n = 7–16 per group. Sal, saline; DEP, diesel exhaust particles; NE, no exercise; E, exercise; RT, room temperature; WT, wild type.

Neutrophil elastase (NE) and double stranded DNA (dsDNA) were assessed as markers for neutrophil extracellular traps (NETs) in BAL. WT DEP/E(/4°C) mice showed significantly more NE in BAL compared to WT DEP/NE(/RT) and Cpa3^cre/+^ DEP/E(/4°C) mice (**Figure 5A**). This effect was not seen in doxantrazole-treated animals compared to resting nor placebo-treated animals (**Figure 5B**). Levels of dsDNA did not differ significantly between groups in either the Cpa3^cre/+^ knockout or the doxantrazole experiment (**Supplementary Figures S5A, B**).

**Figure 6:**
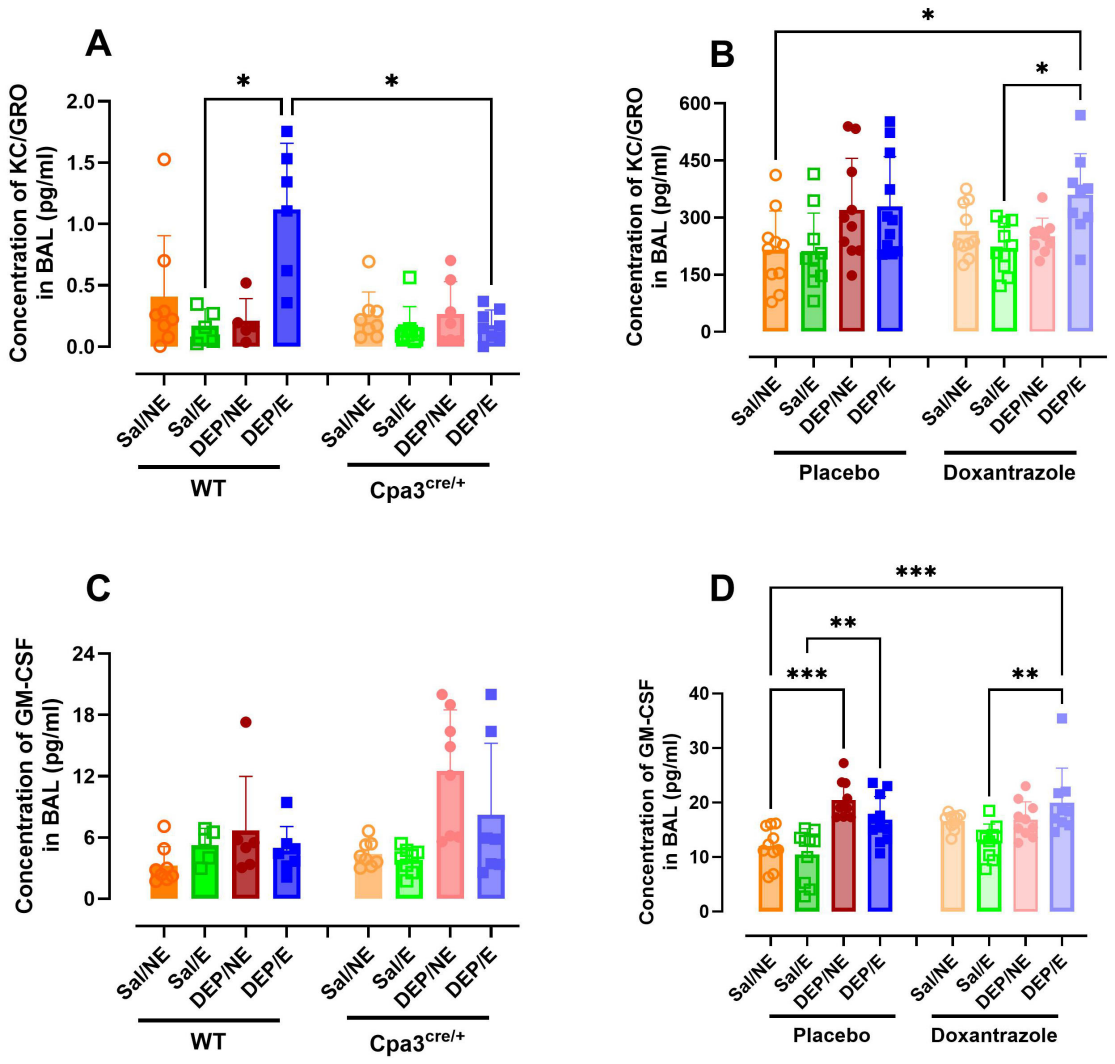
Effect of mast cell inhibition on inflammatory cytokine and chemokine response in the airways to cold air, exercise and diesel exhaust particles. After the experimental running protocol (**Supplementary Figure S1**), animals were sacrificed and bronchoalveolar lavage fluid (BAL) was collected to evaluate airway inflammation through cytokine concentration. Concentration of keratinocyte-derived chemokine (KC) **(A)** and granulocyte-macrophage colony-stimulating factor (GM-CSF) **(C)** present in the BAL of Cpa3^cre/+^ mice compared to WT mice and of KC **(B)** and GM-CSF **(D)** placebo- compared to Doxantrazole treated mice. Data are represented as mean ± SD with individual values and were evaluated using Kruskal–Wallis test with Dunn’s *post hoc* testing. Levels of significance were *p < 0.05, **p < 0.01, ***p < 0.001. n = 7–16 per group. Salll, saline, DEP, diesel exhaust particles, NE, no exercise; E, exercise; RT, room temperature; WT, wild type.

The concentration of different cytokines and chemokines was assessed in BAL fluid. Concentration of keratinocyte chemoattractant (KC) was significantly higher in WT DEP/E(/4°C) mice compared to WT Sal/E(/4°C) and Cpa3^cre/+^ DEP/E(/4°C) mice (**Figure 6A**). No differences were seen between Cpa3^cre/+^ groups (**Supplementary Figure S6**). Similarly, DEP/E(/4°C) mice in the doxantrazole experiment had a significantly higher concentration of KC in the BAL fluid compared to placebo-treated Sal/NE(/RT) and Sal/E(/4°C) mice (**Figure 6B**). However, in this experiment, no differences were seen between placebo-treated and doxantrazole-treated DEP/E(/4°C) mice.

Granulocyte-macrophage colony-stimulating factor (GM-CSF) concentration was not influenced by mast cell inhibition. Only in the doxantrazole experiment, a significant increase in GM-CSF concentration was visible after administering DEP. Yet, no difference was seen between placebo- and Doxantrazole treated mice (**Figure 6D**). This difference was not present in the Cpa3^cre/+^ mice and WT littermates (**Figure 6C**).

TNF-α, IL-1β, IL-2, IL-4, IL-6, IL-10, IL-33, and MCP-1 concentrations did not differ between groups in the Cpa3^cre/+^ knockout or the doxantrazole experiment (**Supplementary Table S2**).

### Mast cell inhibition affects macrophage count and function

**Figure 7:**
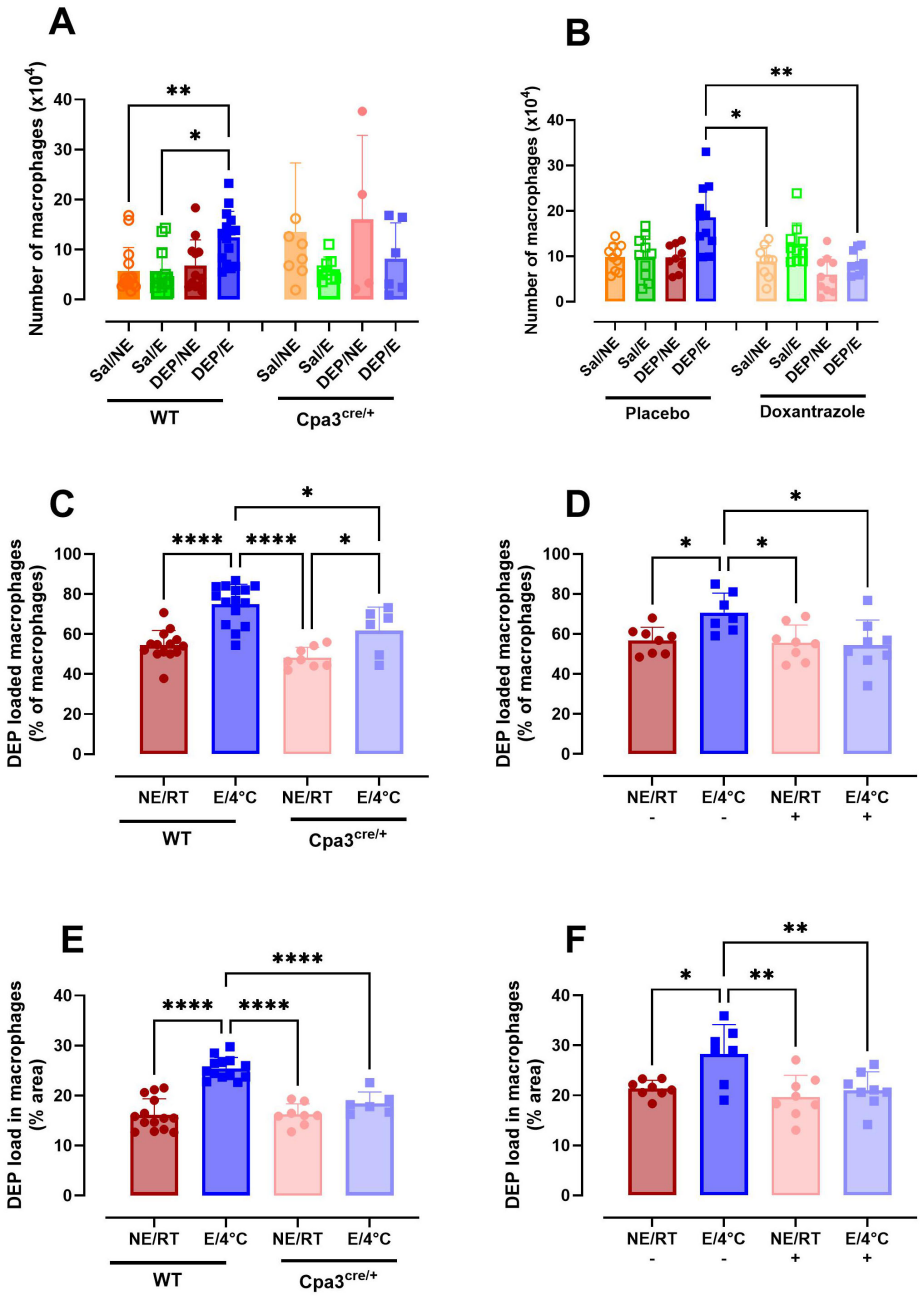
Role of mast cells in macrophage population and particle uptake in the airways in response to cold air, exercise and diesel exhaust particles. After the experimental running protocol (**Supplementary Figure S1**), animals were sacrificed and bronchoalveolar lavage fluid (BAL) was collected to evaluate presence and activity of macrophages. Absolute number of macrophages present in BAL of Cpa3^cre/+^ mice compared to WT mice **(A)** and placebo- compared to Doxantrazole-treated mice **(B)**. The percentage of macrophages loaded with diesel exhaust particles (DEP) as manually counted in Cpa3^cre/+^ mice compared to WT mice **(C)** and placebo- compared to Doxantrazole-treated mice **(D)** and the percentage area of the macrophage covered with particles as calculated with the ImageJ software of Cpa3^cre/+^ mice compared to WT mice **(E)** and placebo- compared to Doxantrazole-treated mice **(F)**. Data are represented as mean ± SD with individual values and were evaluated using Kruskal–Wallis test with Dunn’s *post hoc* testing. Levels of significance were *p < 0.05, **p < 0.01, ****p < 0.001. n = 7–16 per group. Sal, saline; DEP, diesel exhaust particles; NE, no exercise; E, exercise; RT, room temperature; -, placebo; +, Doxantrazole; WT, wild type.

The total number of macrophages did not differ between groups in WT mice and Cpa3^cre/+^ mice. While not statistically significant between Cpa3^cre/+^ and WT mice, the increase in macrophages as seen in WT DEP/E(/4°C) mice was absent in Cpa3^cre/+^ DEP/E(/4°C) mice (**Figure 7A**). In contrast, doxantrazole treatment induced a significantly prevented influx of macrophages compared to placebo in DEP/E(/4°C) mice (**Figure 7B**).

The number of macrophages taking up DEP was significantly higher in WT DEP/E(/4°C) mice compared to non-exercising (RT) mice. Cpa3^cre/+^ mice also had an increased number of macrophages containing particles in their cytoplasm when exercising in cold (DEP/E/4°C) compared to resting Cpa^cre/+^ mice, however the number was still significantly lower compared to WT DEP/E/4°C mice. Doxantrazole treatment prevented particle uptake in the E/4°C group (**Figures 7C, D**). Similar significant differences were seen when evaluating the with DEP covered area of the macrophages (**Figures 7E, F**). Representative image of DEP loading of all groups is available in **Supplementary Figure S7**.

### No effects of mast cells on dendritic subpopulations

No differences were seen in the different subsets of dendritic cells (DC, **Supplementary Table S3**). Wild type mice did not show an increased presence of DC subsets when challenged with DEP and exercise in cold compared to resting saline-challenged mice. Similarly, no differences were seen in the different groups treated with placebo or doxantrazole (data not shown).

## Discussion

This study investigated the role of mast cells in bronchial hyperreactivity and airway inflammation induced by exercise in combination with DEP and cold air in mice. We found that mast cells play an essential role in the development of bronchial hyperreactivity and seem to be involved in the development of neutrophilic inflammation in the lungs.

Cpa3^cre/+^ mice were chosen over other mast cell-deficient mouse models due to their more specific targeting of mast cells. Unlike Kit-dependent models, which affect multiple cell lineages such as erythrocytes and neutrophils (24), Cpa3^cre/+^ mice have more selective depletion of mast cells and basophils. Cpa3^cre/+^ mast cell-deficient mice showed no signs of BHR nor airway inflammation, thereby suggesting that there is a crucial role for mast cells early in the development of exercise-induced bronchoconstriction in association to cold air and DEP exposure. This is in line with the hypotheses that has been summarized by Couto et al., where mast cells are an important player in the development of EIB (3).

Our findings are in line with previous research that showed an important role for mast cells in non-allergic asthma (25), especially in the phenotypes exacerbated by environmental triggers such as cold air and air pollution (26). In allergen-driven models, mast cell deficient mice demonstrated attenuated BHR and inflammation, which could be restored by mast cell reconstitution (27), and mast cell-derived TNF was shown to be critical for BHR development (28).In contrast, we did not observe TNF differences in our model, despite consistent BHR. Other studies reported increased mast cell activation and mediator release in response to air pollution, mainly in an IgE-dependent manner (29–31). Human provocation studies further supported the role of mast cells in airway narrowing, where mast cell-derived prostaglandin D_2_ and leukotriene C_4_ metabolites were increased after mannitol challenges (32). Currently, we were unable to identify the exact mechanism through which mast cells are activated in our model to induce BHR and airway inflammation. Further research with e.g. FcϵRI knockout mice or anti-IgE neutralisation could provide indispensable insights.

To evaluate in which phase of the development of EIB mast cells play a role, WT mice were treated with the mast cell stabilizer doxantrazole, otherwise known as 3-(5-tetrazolyl) thioxanthene-9-one 10, 10-dioxide (33). Although the exact mode of action of doxantrazole remains unclear, it has been suggested that the compound may inhibit 3’-5’-cyclic adenosine monophosphate phosphodiesterase (34), thereby reducing intracellular calcium influx and thus preventing degranulation of mast cells (35, 36). Although doxantrazole is currently not used in clinical practice and may not be the most potent available mast cell stabilizers, its use here was justified based on prior dose optimization and standardized intraperitoneal administration (37). These findings raise the question whether mast cell stabilizers could be used in addition to β2-agonists for the treatment of bronchoconstriction in asthma and EIB. However, further research is required to confirm this in humans.

Doxantrazole treatment was limited to the final week of the experimental to allow development of EIB. Despite the short treatment duration, mast cell stabilization was sufficient to significantly reduce the manifestation of BHR, thereby suggesting that mast cells have an important role in the later stages of EIB and its characteristic bronchial hyperresponsiveness. Yet, the infiltration of neutrophils could not be reversed by doxantrazole treatment, indicating that mast cell inhibition alone does not fully abrogate airway inflammation.

These findings suggest a complex interplay between mast cells, neutrophils and BHR. While mast cells consistently modulate BHR, neutrophilic inflammation appears to be context dependent. This indicates that BHR can occur in the absence and in the presence of neutrophils and that mast cells contributing to BHR partly independent of neutrophil infiltration. Mast cells likely induce bronchoconstriction through the release of early-phase mediators such as histamine and leukotrienes (38). Therefore, the neutrophilia likely represents a parallel or secondary inflammatory process. This is supported by previous studies showing that depletion of neutrophils does not consistently prevent BHR (39). However, future studies investigating direct mast cell-neutrophil interactions *in vitro* or *in vivo* are required to elucidate the mechanisms underlying this complex relationship.

The influence of mast cells on the neutrophils in the airways were further reflected in the neutrophil extracellular traps (neutrophil elastase) and in the concentration of KC/GRO in BAL. Only in the mast cell deficient mice, a significantly lower concentration of neutrophil elastase and KC/GRO in BAL was seen. This difference was not present in the doxantrazole-treated animals. To further elucidate the role of neutrophils in this exposure-associated EIB model, future studies using specific neutrophil depletion strategies or a neutrophil-deficient mouse models should be used. GM-CSF concentrations showed inconsistent increases. In Cpa3^cre/+^ mice and their littermates, cold, exercise, and/or DEP did not affect GM-CSF levels. In contrast, in both doxantrazole- and placebo-treated mice, DEP exposure led to increased concentrations. This pattern suggests that the effect is primarily DEP-induced rather than EIB-related.”

In this study, we showed that macrophages were mildly affected by inhibition of mast cells. Mast cell deficient mice showed a decreased trend in number of macrophages present in the airways. This difference reached significance in doxantrazole-treated mice. Additionally, our findings point to an association between absence of mast cells and lower DEP uptake by macrophages, as well as lower amounts of macrophages taking up DEP. This could indicate that macrophages are less active in the absence of mast cells. Despite that there is no direct link, this aligns with literature showing that macrophages can become more activated in the presence of mast cells (40, 41).

A limitation of this study is the absence of functional mast cell assays, such as a hexosaminidase assay. This would strengthen the mechanistic link between mast cell activation and bronchial hyperresponsiveness and/or airway inflammation. Likewise, macrophage activity could not be objectified with a phagocytotic assay, such as a zymosan uptake assay (42). Future studies should incorporate these analyses to make a stronger mechanistic evaluation of the underlying processes. Additionally, other hypotheses, including neuronal activation (3), should be assessed using inhibitors of TRP-channels or deficient mouse models. In our study, we specifically focused on inflammatory processes detectable in the airway lumen rather than the lung parenchyma. While we assessed neutrophils and NET markers in BALF, we did not perform histological analyses to evaluate neutrophilic infiltration or direct mast cell–neutrophil interactions in lung tissue. Targeting the parenchymal compartment is necessary to define these cellular interactions and to establish a more comprehensive mechanistic link between mast cells and neutrophilic inflammation.

## Conclusion

In conclusion, this study evaluated the role of mast cells in (early) bronchial hyperresponsiveness (BHR) and airway inflammation in an EIB-mouse model exposed to cold air and/or diesel exhaust particles (DEP). We found that mast cells are crucial in the development of BHR and has a complex interplay with neutrophilic inflammation. These findings suggest that mast cells are a promising therapeutic target and may contribute to improved treatment outcomes in patients with exposure-associated EIB.

## Data Availability

The raw data supporting the conclusions of this article will be made available by the authors, without undue reservation.
