# Supplementary material for: Mast cells are essential in the development of exposure-associated exercise-induced bronchoconstriction in a mouse model
Source: Front Immunol. 2025 Oct 15;16:1650057. doi: 10.3389/fimmu.2025.1650057 (PMC12568420; doi:10.3389/fimmu.2025.1650057)
Supplement: Supplementary file 1 [file DataSheet1.docx]

**Supplemental materials**


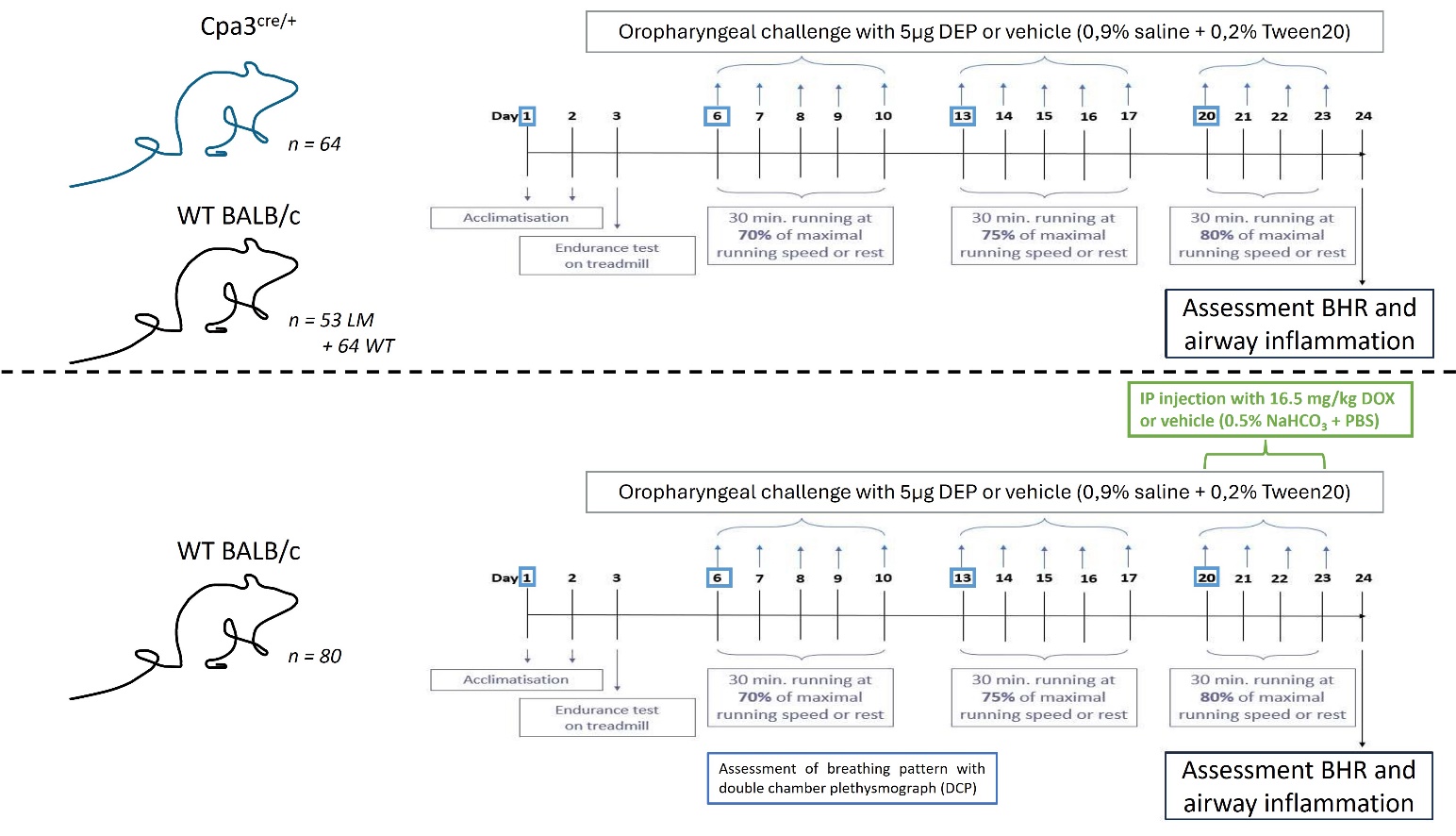


**Figure S1. Experimental study protocol.**


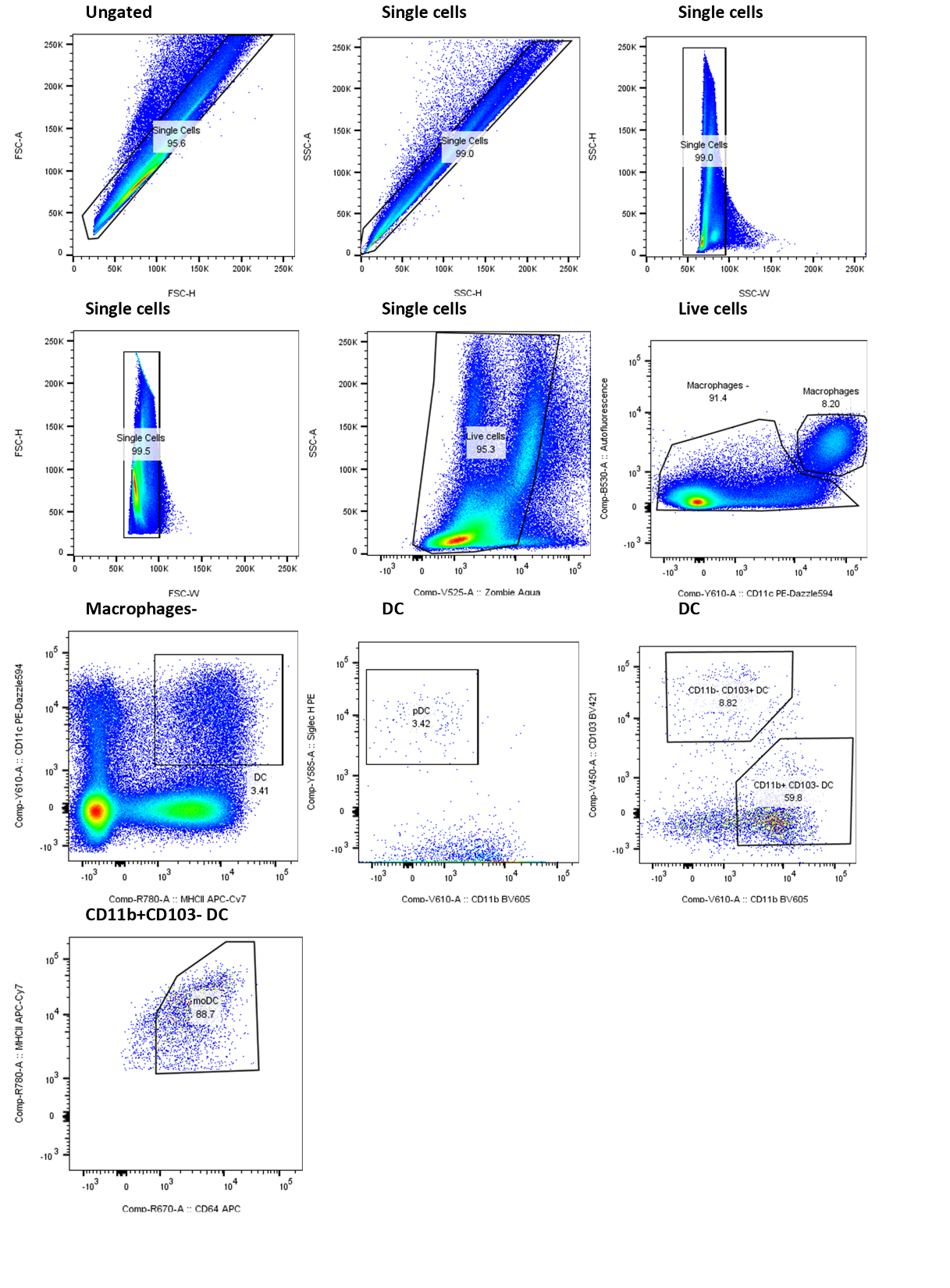


**Figure S2**. **Flow cytometry data and gating for dendritic cell subpopulation in the lung.**

Dendritic cells were marked as CD45+ low auto-fluorescent and CD11c+ and MHCII+. Macrophages were marked as CD45+ high auto-fluorescent and CD11c+. Plasmacytoid dendritic cells (pDC) were gated as CD11b- and Siglec H+. Conventional DCs (cDC) were divided into CD11b-CD103+ cells (cDC1) and CD11b+CD103- (cDC2). Monocyte-derived DCs (moDC) were marked as CD11b+CD103-CD64+. Gating was performed based on the Fluorescence Minus One (FMO) control. Colours represent cell density in the density plot whereby blue represents low density and red represents high density.

**Figure S3. Bronchial hyperreactivity to cold air, exercise and DEP exposure in mast cell deficient mice.**

Dose-response curve in FEV_0.1_ (%) to increasing doses of methacholine (0 – 40 mg/ml) in Cpa3^cre/+^ mice (A). Dose-response curve in airway resistance (Rn) to increasing doses of methacholine (0 – 40 mg/ml) in Cpa3^cre/+^ mice (C). Data was acquired through negative pressure forced expiration (NPFE) maneuver and QP3 forced oscillation technique, respectively, with the flexiVent.

Data are represented as group average and evaluated using two-way ANOVA with Bonferroni multiple comparison post hoc test, n = 7-8 per group. Levels of significance for groups were * compared to Sal/E (4°C, WT [A&C] or placebo [B&D] and # compared to DEP/E (4°C, WT [A&C] or placebo [C&D]). *,# p < 0.05, ** p < 0.01, **** p < 0.0001. n = 7 – 8 per group.

DEP = diesel exhaust particles, NE = no exercise, E = exercise, RT = room temperature, - = placebo, + = Doxantrazole, FEV_0.1_ = forced expiratory volume in 0.1 second, BHR = bronchial hyperresponsiveness.

**Figure S4. Neutrophilic response in the bronchoalveolar lavage fluid of Cpa3^cre/+^ mice.**

Percentual numbers of neutrophils present in the bronchoalveolar lavage fluid of Cpa3^cre/+^ mice. Data are represented as mean ± SD with individual values and were evaluated using Kruskal–Wallis test with Dunn’s post hoc testing. Ln = 5-8 per group.

Sal = saline, DEP = diesel exhaust particles, NE = no exercise, E = exercise, RT = room temperature.

**Figure S5. Respone of mast cell inhibition on neutrophil extracellular traps in the bronchoalveolar lavage fluid.**

Concentration of double stranded DNA (dsDNA) present in the bronchoalveolar lavage fluid of Cpa3^cre/+^ mice compared to WT mice (A) and placebo- compared to Doxantrazole treated mice. Data are represented as mean ± SD with individual values and were evaluated using Kruskal–Wallis test with Dunn’s post hoc testing. Levels of significance were ** p < 0.01. n = 7-16 per group.

Sal = saline, DEP = diesel exhaust particles, NE = no exercise, E = exercise, RT = room temperature, - = placebo, + = Doxantrazole, WT = wild type

**Figure S6. Presence of neutrophil extracellular traps in the bronchoalveolar lavage fluid of Cpa3^cre/+^ mice.**

Concentration of neutrophil elastase (A) and dsDNA (B) present in the bronchoalveolar lavage fluid of Cpa3^cre/+^ mice. Data are represented as mean ± SD with individual values and were evaluated using Kruskal–Wallis test with Dunn’s post hoc testing. Ln = 5-8 per group.

Sal = saline, DEP = diesel exhaust particles, NE = no exercise, E = exercise, RT = room temperature.


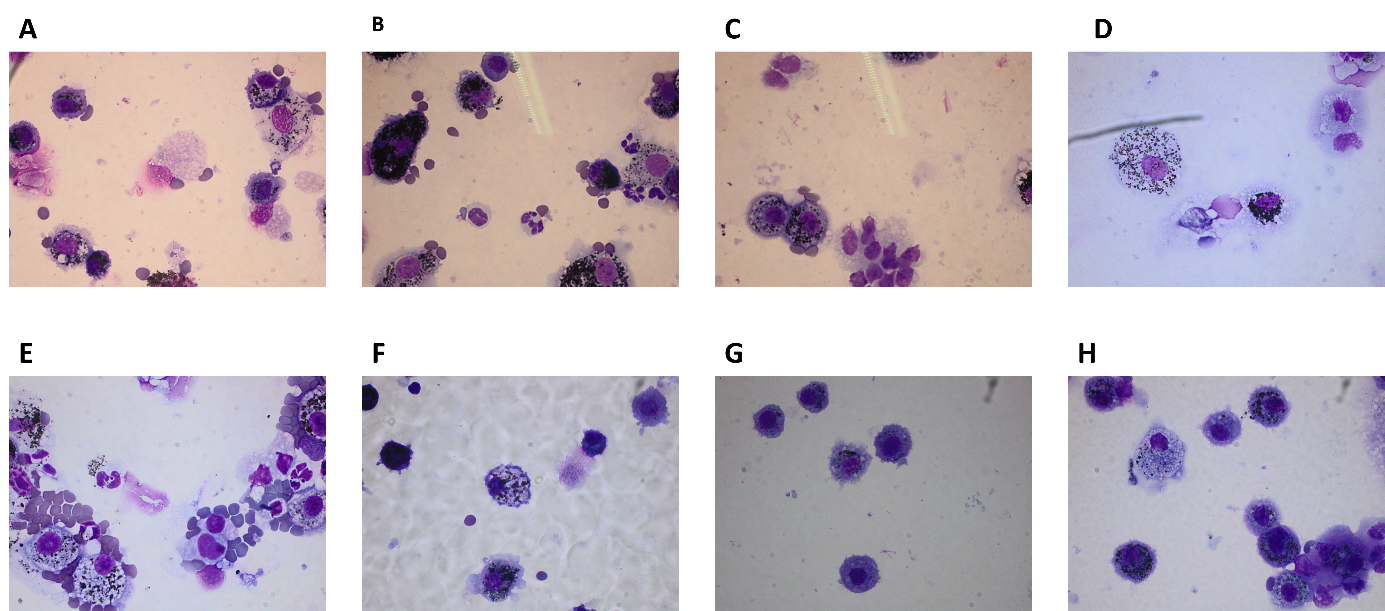


**Figure S7. Representative image of diesel exhaust particle loading in macrophages.**

Representative images of macrophage loading with diesel exhaust particles from DEP/NE/RT WT mice (A), DEP/E/4°C WT mice (B), DEP/NE/RT Cpa3^Cre/+^ mice (C), DEP/E/4°C Cpa3^Cre/+^ mice (D), DEP/NE/RT placebo-treated mice (E), DEP/E/4°C placebo-treated mice (F), DEP/NE/RT doxantrazole-treated mice (G) and DEP/E/4°C doxantrazole-treated mice (H). Magnification 10x.

**Table S1: Overview of used markers for flow cytometric analyses.**

| **Marker** | **Fluorochrome** | **Clone** | **Supplier** | **Catalog number** |
| --- | --- | --- | --- | --- |
| **DC** | | | | |
| CD11c | Pe-Dazzle 594 | N418 | Biolegend | 117348 |
| CD45 | BV711 | 30 – F11 | Biolegend | 103147 |
| CD11b | BV605 | M1/70 | Biolegend | 101257 |
| MHCII | APC-Cy7 | M5/144.15.2 | Biolegend | 107268 |
| CD103 | BV421 | 2E7 | Biolegend | 121422 |
| CD64 | APC | X54-5/7.1 | Biolegend | 139306 |
| Siglec H | PE | 551 | Biolegend | 129606 |

**Table S2: Cytokine measurements in bronchoalveolar lavage fluid.**

Cytokine levels (pg/ml) in bronchoalveolar lavage fluid were measured using U-plex assay (Meso Scale Diagnostics). Data is shown as mean ± standard deviation, n = 5 – 8 per group. GM-CSF = granulocyte-macrophage colony-stimulating factor, IL = interleukin, KC= keratinocyte-derived chemokine, MCP1 = monocyte chemoattractant protein 1, TNF-α = tumour necrosis factor α.

**Table S3: Overview of evaluated dendritic cell subtypes with flow cytometric markers**

| **Cell type** | **Flow cytometric marker** |
| --- | --- |
| Total DC population | CD45^+^ low auto-fluorescence MHCII^+^ CD11c^+^ |
| Plasmacytoid DC | CD45^+^ low auto-fluorescence MHCII^+^ CD11c^+^ SiglecH+CD11b^-^ |
| CD103+ conventional DC | CD45^+^ low auto-fluorescent MHCII^+^ CD11c^+^ CD11b^-^ CD103^+^ |
| Monocyte derived DC | CD45^+^ low auto-fluorescent MHCII^+^ CD11c^+^ CD64^+^ |
| CD103- conventional DC | CD45^+^ low auto-fluorescent MHCII^+^ CD11c^+^ CD11b^+^ CD103^-^ |
